# Alisma Shugan Decoction attenuates hepatic fibrosis and endoplasmic reticulum stress in mice with carbon tetrachloride-induced fibrosis

**DOI:** 10.4314/ahs.v23i2.49

**Published:** 2023-06

**Authors:** Yun-Feng Sun, Hong-Hua Pan, Zhong-Ni Xia, Zhong-Min Yu, Cheng-Le Li, Xiao-Dong Wang, Sheng-Hui Shen

**Affiliations:** 1 Zhejiang Academy of Traditional Chinese Medicine, Hangzhou 310007, Zhejiang Province, China; 2 Department of Pharmacy, Tongde Hospital of Zhejiang Province, Hangzhou 310012, Zhejiang Province, China

**Keywords:** Alisma Shugan Decoction, liver injury, fibrosis, anti-inflammation, unfolded protein response signalling

## Abstract

**Background:**

Over the years, Alisma Shugan Decoction (ASD), because of its potent anti-inflammation activity, has been used in traditional Chinese medicine (TCM) for treatment of many inflammation-associated disorders including those of the heart, blood vessel and brain.

**Methods:**

Herein, we examined the probable therapeutic effect of ASD in carbon tetrachloride (CCl4)-induced liver injury and fibrosis mice models.

**Results:**

Our results demonstrate that ASD dose-dependently reduced the fibrosis-related increased collagen deposition secondary to liver tissue exposure to CCl4. Data from our biochemical analyses showed significantly less liver damage biomarkers including ALT, AST and hydroxyproline in the ASD-treated samples, suggesting hepato-protective effect of ASD. Furthermore, we demonstrated that treatment with ASD significantly reversed CCl4-induced elevation of TNF-α, IL-6, IL-1β and MP-1. Interestingly, NF-κB signalling, a principal regulator of inflammation was markedly suppressed by ASD treatment. In addition, treatment with ASD deregulated stress signalling pathways by suppressing the expression of markers of unfolded protein response, such as ATF6, IRE and GRP78.

**Conclusion:**

In conclusion, the present study provides preclinical evidence for the use of ASD as an efficacious therapeutic option in cases of chemical-induced liver damage and/or fibrosis. Further large-cohort validation of these findings is warranted.

## Background

Globally, Liver cirrhosis, an end stage liver disorder, is clinically challenging due to lack of effective therapies. In the development of liver cirrhosis, hepatic fibrosis is an important step 1, thus, necessitating the discovery or development of therapeutic agents that prevent or reverse fibrosis. As a pathological process, hepatic fibrosis initiates the disruption of normal live architecture by enhancing the deposition of extracellular matrix (ECM) as part of the healing process initiated in response to continuous injury to the liver tissue Hepatic fibrosis is characterized by the trans-differentiation of hepatic satellite cells (HSCs) to α-smooth muscle actin (α-SMA)-positive myofibroblast cells with enhanced synthesis of ECM components 2. Cumulative evidence suggests that hepatic fibrosis may be reversed by removal of the underlying etiological factor, thus building a case for the development of novel antifibrosis therapeutic approaches 3, especially as effective clinical therapies that prevent, halt or reverse fibrosis are still lacking.

Endoplasmic reticulum (ER), an essential organelle required for cell survival and normal cellular function, serve as site for chaperone-assisted folding of nascent proteins. The disbalance between nascent proteins in the ER and ER chaperone reserve results in ER stress, while the accumulation of unfolded and misfolded proteins in the ER lumen causes the dramatic activation of the unfolded protein response (UPR) signalling 4. The UPR signalling pathway is mediated by three transmembrane ER proteins, namely, activating transcription factor (ATF)-6, inositol requiring ER-to-nucleus signal kinase (IRE)-1, and double-stranded RNA-activated kinase (PKR)-like ER kinase (PERK) 5. The critical role of ER stress in the pathogenesis of liver disease is increasingly documented 6. Recently, hepatic ER stress and the activation of UPR signalling were associated with exposure to bile acids in a cholestasis-induced hepatic fibrosis model 7. Similarly, carbon tetrachloride (CCl4)-induced hepatic steatosis or hepatic fibrosis have been associated with ER stress 8 or UPR signalling 9, respectively.

Alisma Shugan Decoction (ASD), a classical traditional Chinese formula from the Eastern Han dynasty, contains the bioactive components Alisma plantago-aquatica L. and Atractylodes macrocephala Koidz., and has been in use for about 1300 years in TCM treatment of liver diseases in Asia, especially in China where it has long been used in the treatment of hepatic fibrosis and other liver pathologies 10. There are studies demonstrating the anti-lipidemic, anti-atherosclerotic and other pharmacological effects of ASD 11. In our previous studies, we showed the limited inhibitory effect of a crude extract of ASD on hepatic fibrosis in murine models. However, the effect of ASD on hepatic fibrosis and ER stress in mice with CCl4-induced fibrosis, as well as its underlying molecular mechanism remain unknown.

This current study investigates the effect of ASD on the liver function and phenotype of mice bearing CCl4-induced hepatic fibrosis. For the first time, to the best of our knowledge, we show that ASD targets pathologic liver inflammatory response and ER stress activation to inhibit CCl4-induced hepatic fibrosis development in the mice. Our results provide some elucidation and mechanistic insight into the therapeutic effects of ASD on ER stress-related hepatic fibrosis and highlight the protective effect of ASD in the fibrotic response to chronic liver injury.

## Materials and methods

### Preparation of water extract from Alisma Shugan Decoction (ASD)

Alisma Shugan Decoction (ASD) is a classical traditional Chinese formula that was first prescribed in the Eastern Han Dynasty, which consists of a combination of two herbs, including *Alisma plantago-aquatica L*. and *Atractylodes macrocephala Koidz.*, Crude materials of Alisma Shugan Decoction (ASD) were commercialized supply and were carefully identified. ASD crude materials were soaked in water for 30 min, mixed in proportion and then decocted twice by water refluxing at 1:6 and 1:4, w/v, respectively for 1 h. The filtrates were then combined and condensed, before being stored at 4°C until use.

### ASD treatment, in vivo studies

6-8 weeks old male C57BL/6J mice (n = 24) weighing 20 – 25 g were used in this study. The mice were acclimated at 22± 2°C with 55±5% humidity under controlled 12:12 h light-dark cycle for at least a week prior to end of study and humane sacrifice of the mice. Mice were well nurtured and allowed access to chow and water *ad libitum*. Mice in the treatment group (n = 8) were injected intraperitoneally with CCl_4_ at 5 µL/g body weight (10% CCl4 in corn oil) twice a week for 4 or 6 weeks. ASD, dissolved in absolute ethanol and diluted further in saline until the final concentration of ethanol was 2.5%, was administered by intraperitoneal injection at 5 or 10 mg/kg/day, starting 2 weeks after initiation of CCl4 treatment. Mice injected with corn oil only (n = 8) or ASD only (n = 8), served as negative controls. The mice were anesthetized with ketamine/xylazine cocktail, sacrificed by CO2 euthanasia at the end of the study and grown tumors were harvested and weighed. Livers were harvested 24 hours after the last injection of CCl4. The study was approved by the Research Ethics Committee (No. LYY18H280005).

### Biochemical parameters

The serum levels of total bilirubin (TBIL) (BC5185, Solarbio, Beijing, China), direct bilirubin (DBIL) (BC5175, Solarbio, Beijing, China), aspartate transaminase (AST) (ab105135, Abcam, US), alanine aminotransferase (ALT) (BC1555, Solarbio, Beijing, China), total bile acid (TBA) (ab239702, Abcam, US), and liver hydroxyproline were measured using commercially available assay kits according to the manufacturer's instructions.

### Tissue histology and tissue block generation

Liver tissues fixation was done using in 4% formalin, followed by embedment in paraffin according to standard procedure. 5 µm thick paraffin-embedded tissue blocks were stained with hematoxylin and eosin (H & E) for morphological analysis. The degree of necrosis was determined based on the mean of 12 randomly-selected fields of view per slide and classified on a scale of 0–3, representing normal, mild, moderate, and severe, respectively. Inflammatory cells in 12 randomly-selected fields were counted under microscope at × 400 magnification. Liver fibrosis was quantified using the Picro-Sirius red stain kit for connective tissue staining (ab150681, Abcam plc, Cambridge, UK). 10 µm sections were mounted on glass slides, de-paraffinized, rehydrated, and then incubated with aqueous solution of saturated picric acid containing 0.1% fast green FCF and 0.1% direct red 80, at room temperature (RT) for 2 h. Morphology of collagen fibers was captured with a light microscope equipped with a charge-coupled device (CCD) digital camera. Morphometric analysis of fibrosis was performed using five random low-power images per animal at 100× magnification.

### Measurement of TNF-α, IL-6 and MCP-1 Levels

The levels of tumor necrosis factor (TNF)-α (MTA00B, R&D Systems, Minneapolis, MN), interleukin (IL)-6 (M6000B, R&D Systems, Minneapolis, MN) and monocyte chemotactic protein (MCP-1) (MJE00B, R&D Systems, Minneapolis, MN) in the liver tissues and serum were measured using ELISA kits according to the manufacturer's instructions. The expression levels were normalized to the value for the control.

### Quantitative Real-Time PCR (qRT-PCR)

Total RNA was obtained from frozen mouse liver using Trizol reagent (Life Technologies, Carlsbad, CA, USA), the residual genomic DNA (gDNA) removed by incubation with RNase-free DNase, and RNA integrity confirmed by formaldehyde gel electrophoresis. Quantification was performed using NanoDrop1000 spectrophotometer (Thermo Fisher Scientific, Waltham, MA, USA). Total RNA (1 µg) was reverse-transcribed and mRNA level was determined by RT- PCR using SYBER Green I Master (Roche Diagnostics GmbH, Mannheim, Germany). Relative changes in mRNA expression levels were determined using the qRT-PCR. The cycle number at which the transcripts were detectable [Cq (Ct)] was normalized to the cycle number of GADPH mRNA detection.

### Western blot analysis

For Western blot analysis, liver tissue was homogenized in 1 mL RIPA buffer containing protease and phosphatase inhibitor cocktails at 4°C. The homogenate was incubated on ice for 30 min and centrifuged at 13,000 g for 30 min at 4°C. The supernatant fraction was obtained and stored at -80°C in aliquots until use. Protein concentration was measured using the Pierce BCA Protein Assay Kit (Thermo Fisher Scientific, Waltham, MA, USA) according to manufacturer's instruction. Equal amounts (30 µg) of protein were separated by 9–12% sodium dodecyl sulfate polyacrylamide gel electrophoresis (SDS-PAGE) and transferred to polyvinylidene difluoride (PVDF) membranes (Millipore, Bedford, MA). The membranes were blocked with 5% skimmed milk in Tris-buffered saline containing 0.05% Tween-20 (TBST) for 30 min at 37°C and incubated overnight at 4°C with the following primary antibodies: anti-BiP/GRP78 (sc-13968, Santa Cruz Biotechnology, Santa Cruz, CA), anti-IRE1 (sc-390960, Santa Cruz Biotechnology), ATF6 (sc-166659, Santa Cruz Biotechnology), peIF2α (sc-12412, Santa Cruz Biotechnology), Bax (sc-20067, Santa Cruz Biotechnology) and Bcl-2 (sc-509, Santa Cruz Biotechnology) antibodies at 1:200-1:1000 dilution with PBST containing 2.5% skimmed milk. Anti-β-actin polyclonal antibody (1:2000; Sigma) was used as loading control. After washing carefully with TBST 3 times, the membranes were incubated with secondary HRP conjugated antibody (1:5,000) (HA1001 and HA1006, HUABIO, Hangzhou, China) for 1 h at RT and visualized using enhanced chemiluminescence (ECL) detection kit (GE Healthcare, RPN2108, Sigma, Merck KGaA, Darmstadt, Germany).

### Immunohistochemical (IHC) staining assay

Immunohistochemistry was performed using polyclonal antibodies for a Bax, Bcl-2, NF-κB, p-IKKα and GRP78 as markers of activated hepatic fibrosis and ER stress, respectively. After recovery of tissue samples, they were fixed with 10% buffered formalin, and paraffin-embedded. 4 µm sections were de-paraffinized, rehydrated in graded ethanol, and then cooked in 25 mM citrate buffer at pH 6.0 in a pressure cooker for 10 min, before being transferred into boiling de-ionized H2O, and allowed to cool for 20 min. For inactivation of endogenous peroxidase activity, the tissue sections were treated with 3% hydrogen peroxide (H2O2), followed by incubation of the slides with primary antibodies against Bax, Bcl-2, NF-κB, p-IKKα, GRP78 and pIRE1α (Santa Cruz Biotechnology, Santa Cruz, CA) at a working dilution of 1:200 overnight at 4°C. Thereafter, the tissue sections were incubated with biotin-labeled secondary antibody and HRP-conjugated streptavidin at RT for 15 min. Then the DAB Kit (ZSGB Biotechnology, Beijing, China) was used for colour development, before the sections were counterstained with hematoxylin, dehydrated, cleared, and mounted on glass slides. Areas of staining were analysed by ImageJ IHC detection software (https://imagej.net/) from ten randomly-selected visual field of the histological section.

### Statistical analysis

All data are expressed as mean ± standard error (SE). The student's t-test was used to test for differences between experimental groups. Statistical analyses were performed using MS Excel v. 2013 (Microsoft, Redmond, WA, USA). p-value < 0.05 was considered to be statistically significant. “n” refers to the sample size or number of mice.

## Results

### ASD inhibits CCl4-induced histological changes and collagen deposition in mice liver injury models

**Figure 1:**
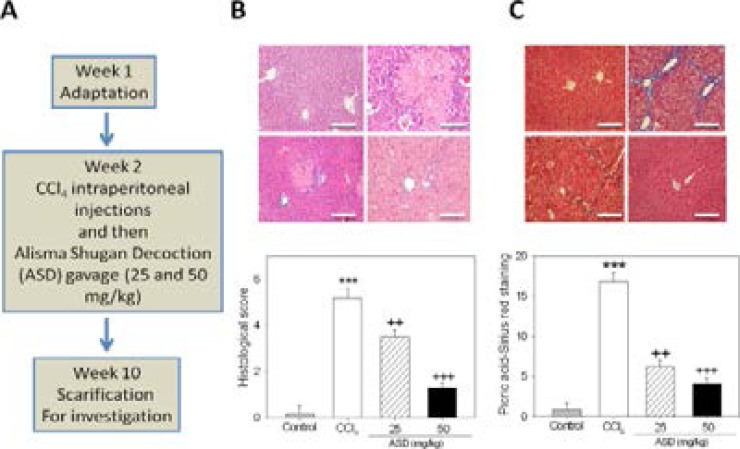
ASD inhibits CCl4-induced histological changes and collagen deposition in mice liver injury models. (A) Schema of the study design. (B) Hematoxylin and eosin staining of liver sections from CCl4 induced liver injury mice models, with their histological score. (C) Representative images and quantification of collagen accumulation as measured by Picro Sirius red staining. Scale bar = 100 µm. Values represent the mean ± S.E (n=10). ** p< 0.01, and *** p < 0.001 vs. Con; + p < 0.05, ++ p < 0.01 and +++ p < 0.001 vs. CCl4 induced liver injury mice models; Mod, CCl4 treatedroup; Con, control group

**Supplementary Figure S1:**
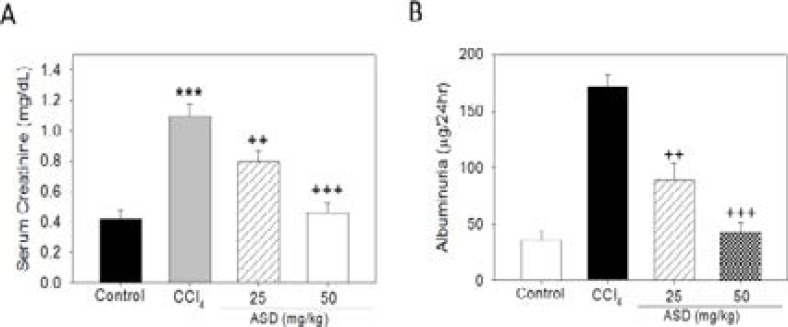
Effects of ASD on renal function of mice. Compared to the control group, ASD group showed (A) lower serum creatinine and (B) reduced 24 h albuminuria. All data are representative of experiments performed 4 times and expressed as means± SD. **p < 0.01 vs. control. ++ p < 0.05, +++ p < 0.01 vs. CCl4 group

In order to investigate the effects of ASD on hepatic damage, male mice were subjected to a 10-week treatment with CCl_4_ with or without 25 or 50 mg/kg ASD (Figure 1A). H&E staining indicated that CCl4 treatment induced liver injury, resulting in higher histological score compared to those in the control group. The administration of ASD ameliorated CCl4-induced liver injury with resultant lower histological score compared with those in the CCl4-treated group (Figure 1B). Moreover, ASD reversed the enhanced collagen accumulation induced by prior treatment with CCl4. Finally, Picro-Sirius Red staining (Abcam plc, Cambridge, UK) revealed marked fibrosis in the CCl4-treated group. However, ASD reduced the fibrotic area in the liver tissue of CCl4-treated mice (Figure 1C). Changes in renal function of aging mice were measured, before and after ASD treatment, and the results were compared with control mice. Serum creatinine was decreased in ASD-treated mice, compared with the control group mice. Twenty-four-hour albuminuria was significantly decreased in the ASD-treated group, compared with the control group (Supplementary Figure S1). These results at least partially indicated that the administration of ASD significantly attenuates chemical-induced liver damage and reduces albuminuria and, thus, improves kidney function in mice.

### ASD reverses liver injury induced by CCl4, in vivo

**Figure 2:**
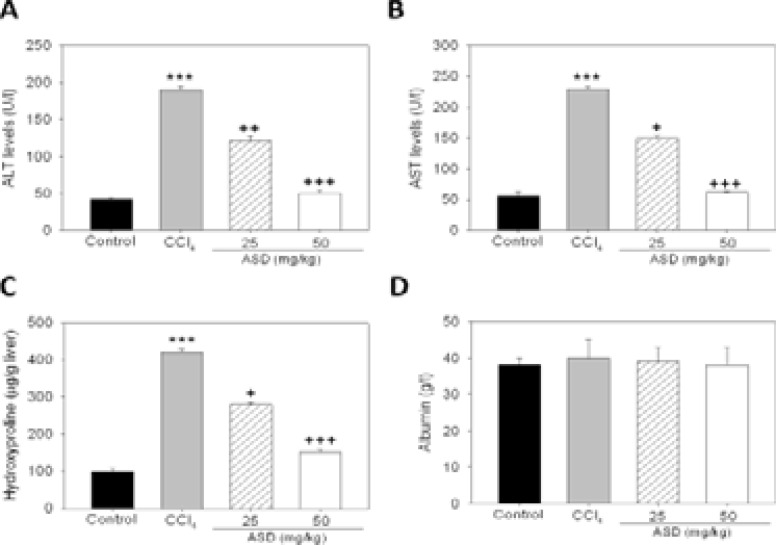
ASD reverses liver injury induced by CCl4, in vivo. Histograms showing the effect of ASD on the production of (A) ALT, (B) AST, (C) Liver hydroxyproline, and (D) serum albumin levels in the CCl4 induced liver injury mice models. Values are expressed as the mean ± standard error of the mean (n=10). Values represent the mean ± S.E (n=10). *** p < 0.001 vs. Con; ++ p < 0.01 and +++ p < 0.001 vs. CCl4 induced liver injury mice models; Mod, CCl4 treated group; Con, control group; ALT, alanine aminotransferase; AST, aspartate aminotransferase

For characterization of the therapeutic effect of ASD on liver injury induced by CCl4, changes in serum ALT, AST, albumin and liver hydroxyproline levels were evaluated. Compared with mice in the control group, the serum ALT and AST levels in the CCl4 group were markedly elevated; conversely, 25 and 50 mg/kg ASD significantly decreased serum ALT and AST levels in dose-dependently (p<0.05) (Figures 2A and 2B). Similarly, ASD reversed the level of CCl4 enhanced hydroxyproline levels in the liver of CCl4 treated mice (Figure 2C). We however observed no significant difference in the liver albumin index, between the four groups (Figure 2D). These results are indicative of the hepato-protective and reparative effects of ASD in subjects with liver injury caused by treatment of CCl4.

### ASD reverses CCl4-induced liver injury by targeting pro-inflammatory cytokine secretion

**Figure 3:**
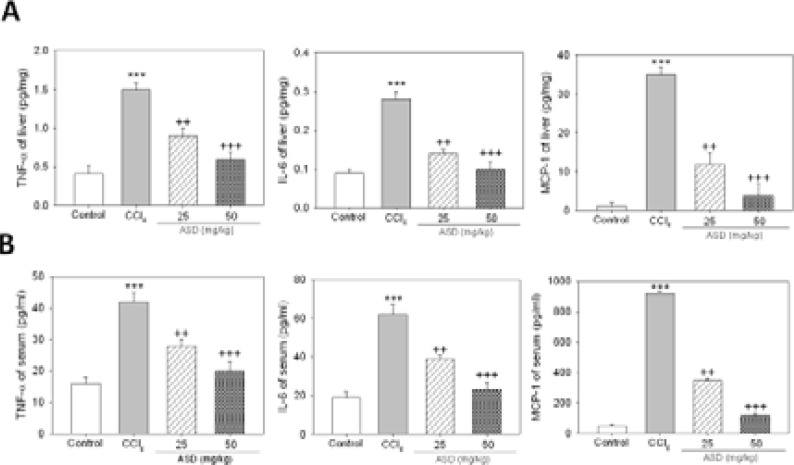
ASD reverses CCl4-induced liver injury by targeting pro-inflammatory cytokine secretion. Graphical representation of the effect of ASD on the (A) expression of TNF-α, IL-6 and MCP 1 in liver tissue, and (B) secretion of serum TNF α, IL-6 and MCP 1, as determined by ELISA. Values represent the mean ± S.E (n=10). ** p< 0.01, and *** p < 0.001 vs. Con; + p < 0.05, ++ p < 0.01 and +++ p < 0.001 vs. CCl4 induced liver injury mice models; Mod, CCl4 treated group; Con, control group; TNF, tumor necrosis factor; IL, interleukin; MCP, monocyte chemotactic protein

Following our results indicating that ASD reverses liver injury induced by CCl4, in vivo, we sought to unravel the underlying mechanism. We observed that exposure to CCl4 strongly upregulated the liver tissue TNF-α, IL-6 and MCP-1 protein levels, but treatment with ASD significantly repressed this upregulated cytokine secretion trend as determined by ELISA (p<0.05) (Figure 3A). Similar results from ELISA assessment of circulating TNF-α, IL-6, and MCP-1, demonstrate that the elevated serum levels of TNF α, IL-6, and MCP-1 in the CCl4-treated mice, were markedly suppressed by ASD (p<0.05) (Figure 3B). ASD showed dose-dependent effect both in liver tissue and in serum. Taken together, these results indicate that ASD suppresses chemical-induced secretion of pro inflammatory cytokines in the serum and their expression in injured liver tissues.

### ASD inhibits liver cell death associated with CCl4-induced liver injury, in vivo

**Figure 4:**
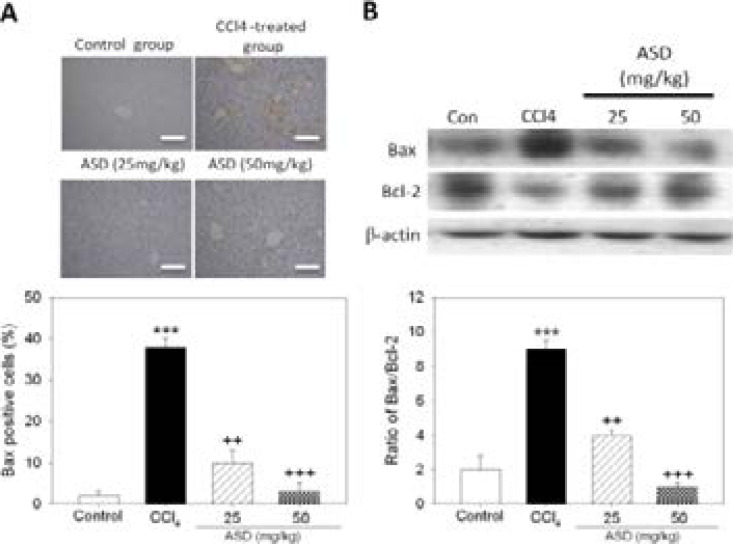
ASD inhibits liver cell death associated with CCl4-induced liver injury, in vivo. (A) IHC image showing the effect of ASD on Bax protein expression in the liver sections. Scale bar = 100 µm. (B) The effect of ASD on the expression level of Bax and Bcl 2 protein as evaluated by western blot analysis (upper panel). Graph showing changes in the Bax/Bcl 2 ratio after treatment with ASD. Values represent the mean ± S.E (n=10). *** p < 0.001 vs. Con; + p < 0.05, ++ p < 0.01 and +++ p < 0.001 vs. CCl4 induced liver injury mice models; Mod, CCl4 treated group; Con, control group; Bcl 2, B cell lymphoma 2; Bax, Bcl 2 associated X protein; PARP, poly (ADP ribose) polymerase

In parallel assays, we assessed the effect of ASD on CCl4-induced liver apoptosis based on the expression level of apoptosis-associated proteins. Exposure to CCl4 markedly increased the mRNA levels of the pro-apoptotic mitochondrial protein Bax, however, this increased Bax mRNA expression was abrogated after the mice were treated with ASD (Figure 4A). In addition, results of our western blot analysis indicated that converse to the expression of Bax, the expression level of the anti-apoptotic Bcl-2 protein was downregulated in the CCl4-treated mice, suggesting that CCl4 induced apoptosis in the liver tissues. Notably, treatment with ASD induced significant downregulation of the Bax/Bcl-2 ratio, thus inhibiting apoptosis (Figure 4B). These results indicate that ASD attenuates CCl4-induced liver injury, in part by suppressing the apoptotic response in the liver tissue.

### ASD reverses CCl4-induced liver injury by deactivation of the NF-κB/p65 signalling pathway

**Figure 5:**
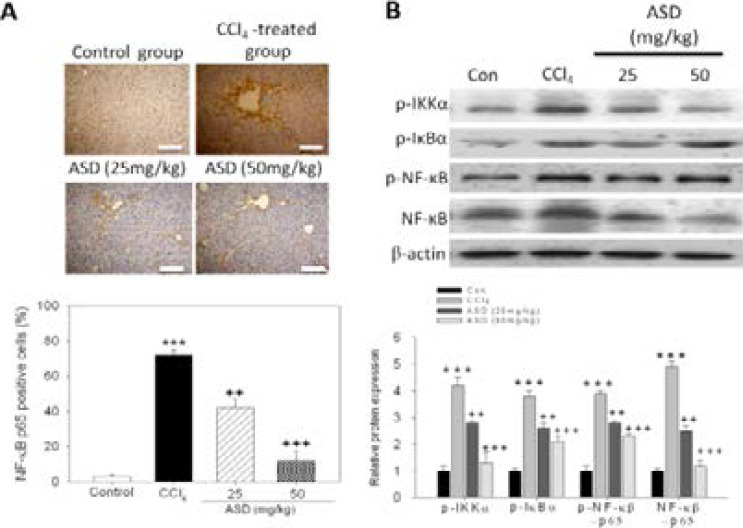
ASD reverses CCl4-induced liver injury by deactivation of the NF-κB/p65 signalling pathway. (A) Photo-image (upper panel) and graph (lower panel) of ASD-induced changes in NF-κB p65 protein expression level in the liver sections of mice after CCl4 induction. Scale bar = 100 µm. (B) The effect of ASD on the expression level of p-IKKα, p-IκBα and p-NF-κB proteins, as determined by western blot analysis. Values represent the mean ± S.E (n=10). *** p < 0.001 vs. Con; + p < 0.05, ++ p < 0.01 and +++ p < 0.001 vs. CCl4 induced liver injury mice models; Mod, CCl4 treated group; Con, control group; p NF κB, phosphorylated nuclear factor κB; IκBα, inhibitor of NF-κB; IKKα, IκB kinase α

To further understand the mechanism underlying the pharmacologic activity of ASD; because of the implication of NF-κB/p65 signalling in course and degree of inflammatory processes, we evaluated the probable effect of ASD on NF-κB/p65 signalling in the mice models of CCl4-induced liver injury using IHC and western blot analyses. Our IHC staining data indicated that relative to the control group, NF-κB/p65 is significantly upregulated in the liver tissue samples of mice exposed to CCl4 (Figure 5A, upper panel). Conversely, the percentage of NF-κB/p65 positive cells was markedly lower in liver tissue samples from the ASD-treated mice, compared to in the CCl/span>4-treated group (Figure 5A, lower panel). Furthermore, we evaluated the effect of ASD on components of the NF-κB signalling pathway. Our results showed that, concurrent with significantly higher level of phosphorylated NF-κB (p-NF-κB), the mice exposed to CCl4 also exhibited higher levels of phosphorylated IKKα (p-IKKα) and IκBα (p-IκBα) protein, compared to the control group (Figure 5B). Interestingly, IKKα, IκBα and NF-κB phosphorylation/activation was significantly downregulated in the ASD-treated mice pre-exposed to CCl4, compared to mice treated with CCl4 alone (Figure 5B). These results demonstrate that the administration of ASD deactivates the NF κB/p65 signalling pathway in the mice model of CCl4–induced liver injury.

### ASD alleviates CCl4-induced hepatic ER stress and deactivates UPR signalling in CCl4-induced liver injury

**Figure 6:**
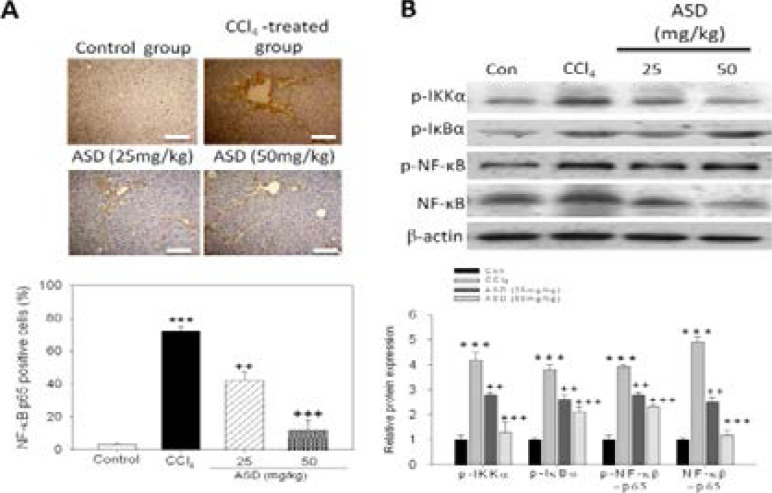
ASD alleviates CCl4-induced hepatic ER stress and deactivates UPR signalling in CCl4-induced liver injury. Mice were intraperitoneally injected with CCl4 (0.15 ml/kg BW, twice per week) in combination with ASD (25 and 50 mg/kg, twice per day) for 8 weeks. Representative photo-image and graphical representation of the effect of ASD on the protein expression levels of hepatic (A) GRP78, (B) nuclear ATF6, (C) p-eIF2α and (D) p-IRE1α as detected by western blot analysis. All proteins were normalized to α-tubulin or lamin A/C level in the same samples. All data are representative of experiments performed 4 times and expressed as means± SD. **p < 0.01 vs. control. ++ p < 0.05, +++ p < 0.01 vs. CCl4 group

Consistent with results above, we also demonstrated that ASD significantly repressed the CCl4-enhanced expression of hepatic ER chaperone GRP78 in mice models of CCl4-induced liver injury (Figure 6A). Similarly, the level of nuclear ATF6 protein which was elevated in the mice models of CCl4-induced liver injury, was significantly reversed by treatment with ASD, dose-dependently (Figure 6B). In addition, ASD also downregulated the expression levels of hepatic p-eIF2α (Figure 6C) and p-IRE1a (Figure 6D) in the mice bearing CCl4-induced liver injury.

## Discussion

As report, carbon tetrachloride (CCl4) -induced hepatic steelosis or hepatic fiber have been associated with ER stress 8 or UPR signalling 9, carefully. The present study reports for the first time the therapeutic effects of ASD on CCl4-induced hepatic fibrosis and probable underlying molecular mechanism in mice models. The ER Stress-UPR signalling axis plays an important role in fibrogenesis. Using CCl4-induced mice hepatic fibrosis models, we demonstrated significantly elevated values for the liver-function biomarkers, ALT, AST, ALP and TBILin the CCl4 group compared to the control group, and most mice in the CCl4 group had ascites.

Mice in the treatment group, after treatment with the ASD exhibited improved liver function compared to mice in the CCl4 group (p < 0.01; Figure 1) and is consistent with the lowering of hepatitis grade and hepatic fibrosis stage. Using the Picro-Sirius red staining, we also provide evidence indicating ASD effectively inhibit CCl4-enhanced collagen deposition, and consequently shrink the fibrotic area in the liver tissue. Added to the reduction of liver fibrosis, supplementation with ASD also improved the hepatic function in the CCl4 induced liver injury models, as can be inferred by repression of ALT and AST levels which were hitherto elevated by CCl4 induction (Figure 2). These finding are clinically-significant as they highlight the hepato-protective and/or reparative effect of ASD on pre-clinical models of chemical-induced hepatic fibrosis.

This study also provided some mechanistic insight into the therapeutic activity of ASD in subjects with chemical-induced inflammation-associated liver injury and/ or impaired liver function. The inflammatory cascade is broadly implicated in the causation and/or augmentation of stress-related organ injury 12. Treatment with CCl4 has been shown to induce inflammation-associated acute liver injury, with elevation of pro-inflammatory cytokine secretion 13. Consistent with the above, the present study demonstrates highly enhanced production of pro-inflammatory cytokines TNF-α, IL-6 and MCP-1 in mice models of CCl4–induced liver injury, which was effectively inhibited by treatment with ASD (Figure 3), indicating, at least in part, that the ameliorative role of ASD in the pre-clinical models of CCl4-induced liver injury is mediated by the suppression of pro-inflammatory proteins, including TNF-α, IL-6 and MCP-1.

In the last two decades, cumulative evidence indicates that necrosis and/or the apoptosis of hepatocytes are associated with cell death in the centrilobular region of the liver bearing CCl4-induced acute liver injury 14. In concordance with this, the present study showed that exposure to CCl4 disrupted the normal cellular architecture, resulting in the loss of nuclei integrity and increased number of fragmented and condensed nuclei in a mice liver tissue, suggestive of liver apoptosis and/or necrosis. This study also demonstrated that marked reversal of the chemical-induced liver injury by ASD, was associated with upregulated expression of Bcl-2 and downregulated Bax expression (Figure 4). This is clinically-important because B-cell lymphoma (Bcl)-2 which is encoded by the BCL2 gene in humans, is a key regulator and biomarker of apoptosis, as well as Bax, which is an important biomarker of mitochondrial apoptosis and associated with the induction of Caspase-3-mediated apoptosis 15. Thus, these data support the role of ASD as a potential inhibitor of chemical-induced oxidative stress with the consequent mitochondrial dysfunction and liver cell death.

NF-κB is a documented master regulator of gene expression, regulating the expression of ≥500 genes associated with inflammation, tumorigenesis, cell survival and/or proliferation, and chemoresistance 16. In the context of the present study, NF-κB plays a central role in stress-induced inflammatory responses; in fact, the activation of NF-κB in response to LPS induction, has been shown to elicit inflammatory mediators, including TNF-α, IL-18, IL-6 and IL-1β 17. The inhibitor of nuclear factor kappa-B kinase (IKK) complex also activated by LPS through the toll-like receptor (TLR)-4 signalling pathway and phosphorylates IκBα in the cytoplasm. The subsequent proteasomal degradation of IKK leads to NF-κB release and nuclear translocation, activating inflammation-responsive transcripts 18. In line with this, we demonstrated that ASD markedly suppressed NF-κB-p65 protein level which was hitherto upregulated by exposure to CCl4 (Figure 5). Concurrently, ASD also downregulated the expression levels of MCP-1, TNF-α and IL-6 proteins in mice model of CCl4-induced liver injury, dose-dependently.

Cellular autophagy is triggered in response to ER stress through the induction of the UPR 19. The UPR has three proximal effectors: PERK, ATF6, GRP78, IRE1, and peIF2a. UPR sensors in mammalian cells regulate signal-transduction pathways associated with the autophagic gene transcription 20, while the silencing of the BiP, an upstream regulator of UPR, inhibits autophagosome formation 21. We showed that treatment with ASD markedly inhibited the expression of ER stress chaperones GRP78, ATF6, and IRE1in the mice models of CCl4-induced liver injury (Figure 6), inferring probable ASD-inhibition of UPR signalling and autophosphorylation of PERK. This is important as the autophosphorylation of PERK activates/phosphorylates eukaryotic initiation factor 2-alpha (eIF2α) and facilitates the translation of ATF4 and ATF6, which are specific cap-independent cellular ER stress response genes. Our data indicated that ASD reverses CCl4-induced activation of the UPR signalling by repressing the elevated expression of GRP78, ATF6, IRE1, and peIF2α expression associated with fibrogenesis and ER-stress-induced autophagy 22, 23.

## Conclusion

**Figure 7:**
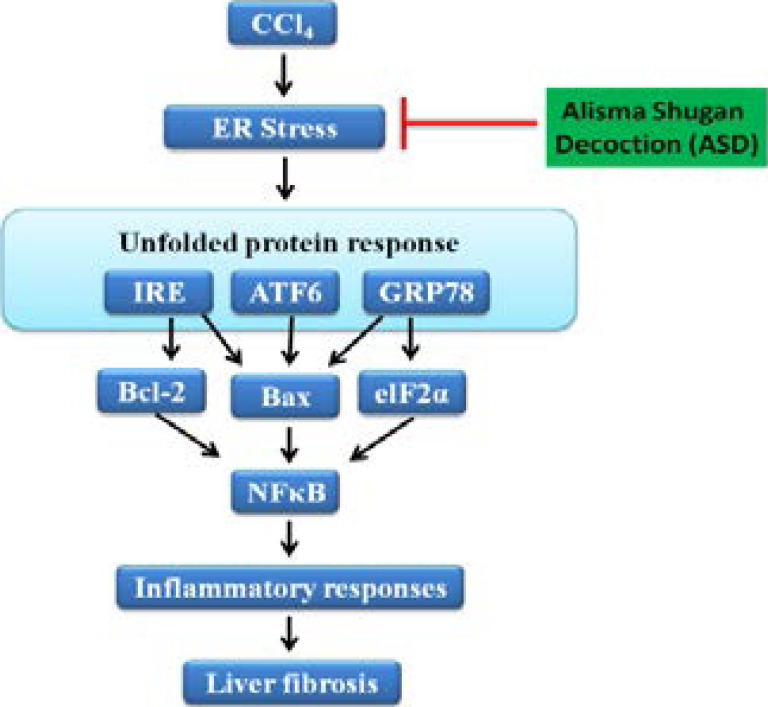
Schematic abstract showing that ASD exhibits strong hepato-protective and reparative effects in chemical-induced liver injury/fibrogenesis. Long-term CCl4 administration induces hepatic ER stress and UPR signalling activation, which are a prelude to CCl4-induced inflammation, and subsequent hepatic fibrosis. Conversely, ASD alleviates CCl4-induced inflammation and hepatic fibrosis by significantly inhibiting CCl4-induced hepatic ER stress, UPR signalling, NF-κB activation and IKKα/β phosphorylation

As shown in the schematic abstract (Figure 7), our findings indicate that ASD exhibits strong hepato-protective and reparative effects, as it alleviates CCl4-induced hepatic ER stress, deactivates UPR signalling, and deregulates the NF-κB signalling pathway. We established a link between this protective function and the inhibition of oxidative stress and inflammatory response. In summary, the current study provides new insights into the hepato-protective effects of ASD and provides a basis for large cohort studies for clinical validation of these findings.

## Abbreviations

TBIL: total bilirubin
DBIL: direct bilirubin
TBA: total bile acid
AST: Aspartate Transaminase
ALT: Liver hydroxyproline and alanine aminotransferase
ATF: activating transcription factor
ASD: Alisma Shugan Decoction
CCl4: carbon tetrachloride
ECM: extracellular matrix
ER: Endoplasmic reticulum
HSCs: hepatic satellite cells
IRE-1: inositol requiring ER-to-nucleus signal kinase
UPR: unfolded protein response
PAGE: polyacrylamide gel electrophoresis
TBST: Tris-buffered saline containing 0.05% Tween-20

## Declarations

### Availability of data and materials

The materials and methods used and/or analysed during the current study are available from the corresponding author on reasonable request.
